# Association of Primary Humoral Immunodeficiencies With Psychiatric Disorders and Suicidal Behavior and the Role of Autoimmune Diseases

**DOI:** 10.1001/jamapsychiatry.2020.1260

**Published:** 2020-06-10

**Authors:** Josef Isung, Kyle Williams, Kayoko Isomura, Caroline Gromark, Eva Hesselmark, Paul Lichtenstein, Henrik Larsson, Lorena Fernández de la Cruz, Anna Sidorchuk, David Mataix-Cols

**Affiliations:** 1 Centre for Psychiatry Research, Department of Clinical Neuroscience, Karolinska Institutet, Stockholm Health Care Services, Region Stockholm, Stockholm, Sweden; 2 Department of Psychiatry, Massachusetts General Hospital, Boston; 3 Department of Psychiatry, Harvard Medical School, Boston, Massachusetts; 4 Department of Medical Epidemiology and Biostatistics, Karolinska Institutet, Stockholm, Sweden; 5 School of Medical Sciences, Örebro University, Örebro, Sweden

## Abstract

**Importance:**

The hypothesis that disrupted immune function is implicated in the pathophysiology of psychiatric disorders and suicide is gaining traction, but the underlying mechanisms are largely unknown. Primary humoral immunodeficiencies (PIDs) are rare deficiencies of the immune system—mainly dysfunction of antibody production—and are associated with adverse health problems, such as recurrent infections and autoimmune diseases.

**Objective:**

To establish whether PIDs that affect antibody function and level are associated with lifetime psychiatric disorders and suicidal behavior and whether this association is explained by the co-occurrence of autoimmune diseases.

**Design, Setting, and Participants:**

This population- and sibling-based cohort study included more than 14 million individuals living in Sweden from January 1, 1973, through December 31, 2013. Register-based data on exposure, outcomes, and covariates were collected through December 31, 2013. Individuals with a record of PID were linked to their full siblings, and a family identification number was created. Data were analyzed from May 17, 2019, to February 21, 2020.

**Exposures:**

Lifetime records of PID and autoimmune disease.

**Main Outcomes and Measures:**

Lifetime records of 12 major psychiatric disorders and suicidal behavior, including suicide attempts and death by suicide.

**Results:**

A lifetime diagnosis of PID affecting immunoglobulin levels was identified in 8378 patients (4947 women [59.0%]; median age at first diagnosis, 47.8 [interquartile range, 23.8-63.4] years). A total of 4776 clusters of full siblings discordant for PID was identified. After adjusting for comorbid autoimmune diseases, PIDs were associated with greater odds of any psychiatric disorder (adjusted odds ratio [AOR], 1.91; 95% CI, 1.81-2.01) and any suicidal behavior (AOR, 1.84; 95% CI, 1.66-2.04). The associations were also significant for all individual psychiatric disorders (range of AORs, 1.34 [95% CI, 1.17-1.54] for schizophrenia and other psychotic disorders to 2.99 [95% CI, 2.42-3.70] for autism spectrum disorders), death by suicide (AOR, 1.84; 95% CI, 1.25-2.71), and suicide attempts (AOR, 1.84; 95% CI, 1.66-2.04). In the sibling comparisons, the associations were attenuated but remained significant for aggregated outcomes (AOR for any psychiatric disorder, 1.64 [95% CI, 1.48-1.83]; AOR for any suicidal behavior, 1.37 [95% CI, 1.14-1.66]), most individual disorders (range of AORs, 1.46 [95% CI, 1.23-1.73] for substance use disorders to 2.29 [95% CI, 1.43-3.66] for autism spectrum disorders), and suicide attempts (AOR, 1.41; 95% CI, 1.17-1.71). Joint exposure for PID and autoimmune disease resulted in the highest odds for any psychiatric disorder (AOR, 2.77; 95% CI, 2.52-3.05) and any suicidal behavior (AOR, 2.75; 95% CI, 2.32-3.27). The associations with psychiatric outcomes (AORs, 2.42 [95% CI, 2.24-2.63] vs 1.65 [95% CI, 1.48-1.84]) and suicidal behavior (AORs, 2.43 [95% CI, 2.09-2.82] vs 1.40 [95% CI, 1.12-1.76]) were significantly stronger for women than for men with PID.

**Conclusions and Relevance:**

Primary humoral immunodeficiencies were robustly associated with psychopathology and suicidal behavior, particularly in women. The associations could not be fully explained by co-occurring autoimmune diseases, suggesting that antibody dysfunction may play a role, although other mechanisms are possible. Individuals with both PID and autoimmune disease had the highest risk of psychiatric disorders and suicide, suggesting an additive effect. Future studies should explore the underlying mechanisms of these associations.

## Key Points

**Question:**

Are primary humoral immunodeficiencies associated with psychiatric disorders and suicidal behavior?

**Findings:**

In this population-based cohort study of 8378 patients in Sweden, having a record of primary humoral immunodeficiencies was associated with greater odds of psychiatric disorders and suicidal behavior, even after controlling for autoimmune diseases and familial confounding. The associations were significantly stronger in women and among those exposed to primary humoral immunodeficiencies and autoimmune diseases.

**Meaning:**

Primary humoral immunodeficiencies were robustly associated with psychiatric disorders and suicidal behavior, particularly in women; the association could not be fully explained by co-occurring autoimmune diseases or by familial confounding, and the mechanisms remain to be elucidated.

## Teaser

This cohort study assesses whether primary humoral immunodeficiencies that affect antibody level and function are associated with lifetime psychiatric disorders and suicidal behavior, and whether this association is explained by the co-occurrence of autoimmune diseases among adults in Sweden.

## Introduction

Mounting evidence suggests that immune disruption may be etiologically important in psychiatric disorders through a range of mechanisms, such as altered neurodevelopment, postinfectious priming of microglia, or microbial dysbiosis.^1,2,3^ However, little is known about the neuropsychiatric consequences resulting from the underproduction of homeostatic antibodies. Thus, primary humoral immunodeficiencies (PIDs)^4^ could provide an interesting model to disentangle the effects of humoral immunodeficiency and autoimmune diseases on psychiatric disorders.

Most PIDs affect antibodies^5^ and are associated with increased risk of recurrent infections as well as with a markedly increased risk of developing autoimmune diseases.^6,7^ Selective IgA deficiency (the most common PID among white individuals)^8^ has been linked to increased infections within the mucosa-associated lymphoid tissue (MALT), an important immune barrier.^8^ In turn, infections within the MALT have long been suspected of association with certain forms of psychopathology in children, particularly obsessive-compulsive disorder and chronic tic disorders.^9^ Preclinical evidence suggests that repeated inoculation of group-A streptococci in the homologous tonsil region of mice leads to T helper 17 cell proliferation, blood-brain barrier breakdown, and inflammation within the brain, even without evidence of bacterial invasion.^10^ A large population-based study showed that chronic inflammation within the MALT (using tonsillectomy as a proxy) was robustly associated with a broad range of psychiatric disorders and suicidal behavior,^11^ suggesting that the association between immune dysfunction and psychopathology may not be uniquely relevant to obsessive-compulsive disorder and tic disorders. In fact, a growing body of evidence suggests that all studied forms of psychopathology are associated with autoimmune disease.^12,13,14,15,16,17,18^

This study aimed to explore whether PIDs that affect immunoglobulins are associated with a broad range of psychiatric disorders and suicidal behavior. Further, we explored the potential contribution of co-occurring autoimmune diseases to the observed associations and used a within-family design to account for shared familial confounders. Given that PIDs are rare disorders, the use of the Swedish nationwide registers provides a unique opportunity to extend our knowledge on the association among immune deficiencies, autoimmune diseases, and psychiatric disorders and suicidal behavior.

## Methods

The study was approved by the regional ethical review board in Stockholm. The board waived the requirement for informed consent because the study was register based and the included individuals were not identifiable at any time. This study followed the Strengthening the Reporting of Observational Studies in Epidemiology (STROBE) reporting guideline.

### Data Sources

Swedish nationwide health and administrative registers were linked using the unique national identification number.^19^ Data on demography and migration were extracted from the Total Population Register and the Migration Register.^20^ Data on kinship were extracted from the Multi-Generation Register,^21^ and causes and dates of death were collected from the Cause of Death Register.^22^ Data covering prescribed and dispensed medications from July 2005 were extracted from the Prescribed Drug Register.^23^ The National Patient Register provided information on diagnoses given in both inpatient (from 1964, with nationwide coverage for psychiatric disorders from 1973) and outpatient (since 2001) specialist services.^24^ Diagnoses are based on the *International Classification of Diseases, Eighth Revision* (*ICD-8*; 1969-1986), *International Classification of Diseases, Ninth Revision* (*ICD-9*; 1987-1996), and *International Statistical Classification of Diseases and Related Health Problems, Tenth Revision* (*ICD-10*; 1997 onward).

### Study Population

A population-based cohort included all individuals living in Sweden anytime from January 1, 1973, to December 31, 2013. Register-based data on exposure, outcomes, and covariates were collected through December 31, 2013. Individuals with a record of PID were linked to their full siblings, and a family identification number was created. A sibling comparison model accounts for unmeasured familial confounders, given that full siblings share a mean of 50% of genetic factors and much of the early environment. Siblings were identified within the same cohort and were included in the family-level analysis if they had at least 1 full sibling discordant for PID.

### Variables

#### Exposure

Individuals with any PID diagnosis affecting immunoglobulin levels ever recorded in the National Patient Register from 1973 to 2013 were considered exposed (see *ICD* codes in eTable 1 in the Supplement). Lifetime exposure to PIDs was dichotomized as any PID vs no PID. In addition, we identified individuals with a lifetime diagnosis of selective IgA deficiency from 1997 to 2013 (*ICD-10* only; eTable 1 in the Supplement). Individuals were considered unexposed if no records of any PID were identified.

We collected lifetime records of autoimmune diseases on specific diagnoses (see *ICD* codes in eTable 2 in the Supplement) from which we constructed a dichotomous variable for any autoimmune disease. Then, we constructed a variable for joint exposure, which was categorized as any PID only, any autoimmune disease only, any PID plus any autoimmune disease, or none.

#### Outcomes

A lifetime record of a psychiatric disorder or a suicide attempt in the National Patient Register (as inpatient or outpatient care) or a record of death by suicide in the Cause of Death Register constituted the outcome. Psychiatric disorders included 12 individual disorders (see diagnoses, *ICD* codes, and median [interquartile range (IQR)] age at the first record of diagnosis in eTable 3 in the Supplement). We created dichotomous variables for each individual disorder and constructed a combined variable for any psychiatric disorder. For suicidal behavior outcomes, we retrieved data on all deaths by suicide and all lifetime records of suicide attempts (see diagnoses, *ICD* codes, and median [IQR] age at the first record of diagnosis in eTable 4 in the Supplement). We constructed separate dichotomous variables for suicide attempts and death by suicide and a combined variable for any suicidal behavior. Minimal age limits were applied for identification of outcomes to reduce the risk of diagnostic misclassification (eTables 3 and 4 in the Supplement).

#### Covariates

Lifetime records of autoimmune diseases (as described above) were considered as a potential confounder. From the Total Population Register, we collected information on the individuals’ birth year and sex.

### Statistical Analysis

Data were analyzed from May 17, 2019, to February 21, 2020. All tests used 2-tailed, unpaired testing, with a significance level set at *P* < .05.

#### Cohort Analyses

For the main analysis, logistic regression models were fitted to estimate odds ratios (ORs) and 95% CIs for the association between lifetime records of PID and the following outcomes: (1) any psychiatric disorder, (2) individual psychiatric disorders, (3) any suicidal behavior, (4) suicide attempts, and (5) death by suicide. A model minimally adjusted for birth year and sex was followed by a model with an additional adjustment for lifetime records of any autoimmune disease.

#### Sibling Analyses

Conditional (fixed-effect) logistic regression models were fitted in the subcohort of full siblings discordant for PID, for which each family was considered a stratum. Within a family, all full siblings were compared with each other, with exposed siblings having their unexposed siblings as controls with the same adjustment strategy as in the cohort analysis.

#### Additional Analyses

Four additional analyses were performed. First, to determine the possible additive effect from multiple disruptions of the immune system, we assessed the effect of joint and single exposure to any PID and any autoimmune disease in comparison with none of these diagnoses. A logistic regression model was applied to the whole study cohort and adjusted for birth year and sex.

Second, we focused on selective IgA deficiency, a less severe PID than common variable immunodeficiency. A logistic regression model was fitted in the subcohort of those living in Sweden from 1997 to 2013, with the same adjustment strategy as in the main analysis. Individuals with PIDs other than selective IgA deficiency were excluded from this analysis. A sibling analysis was conducted by comparing individuals with selective IgA deficiency with their full siblings with no records of any PID.

Third, the analyses for outcomes in association with joint and single exposure to any PID and any autoimmune disorder were stratified by sex. Fourth, to address a potential bias from cases with more severe outcomes, we repeated the main analyses after excluding individuals with an exposure and outcome diagnosed before 2001 (ie, when the National Patient Register records were based solely on inpatients visits). This analysis was conducted for individuals living in Sweden in 2001 onward and based on data from both inpatient and outpatient outcome records to ensure generalizability of the results to patients with less severe exposures or outcomes.

## Results

In the initial cohort of 14 306 315 individuals, we identified 8378 patients (4947 women [59.0%] and 3431 men [41.0%]; median age at first diagnosis, 47.8 [IQR, 23.8-63.4] years) with a record of PID affecting immunoglobulin levels. Among the patients with PID, 2309 (27.6%) had a diagnosis of an autoimmune disease, whereas among the unexposed individuals, 967 774 (6.8%) had a diagnosis of an autoimmune disease, with a statistically significant difference in proportions (*P* < .001). Table 1 reports the descriptive characteristics of the full cohort and sibling subcohort by exposure status.

**Table 1.** Descriptive Characteristics of the Population Cohort and the Full-Siblings Subcohort

Characteristic	Study group, No. %^a^			
	Population cohort		Full-siblings cohort	
	PID exposed (n = 8378)	PID unexposed (n = 14 297 937)	PID exposed (n = 4828)	PID unexposed (n = 8584)
Women	4947 (59.0)	7 078 654 (49.6)	2780 (57.6)	4231 (49.3)
Distribution of years of birth				
1949 or earlier	3665 (43.7)	5 800 579 (40.6)	1370 (28.4)	2744 (32.0)
1950-1959	1082 (12.9)	1 357 472 (9.5)	760 (15.7)	1534 (17.9)
1960-1969	976 (11.6)	1 480 065 (10.4)	718 (14.9)	1173 (13.7)
1970-1979	721 (8.6)	1 436 823 (10.0)	533 (11.0)	891 (10.4)
1980-1989	654 (7.8)	1 383 550 (9.7)	495 (10.3)	860 (10.0)
1990-1999	704 (8.4)	1 242 873 (8.7)	534 (11.1)	792 (9.2)
2000 or later	576 (6.9)	1 596 575 (11.2)	418 (8.7)	590 (6.9)
Age at record of PID, y				
0-9	1183 (14.1)	NA	865 (17.9)	NA
10-19	648 (7.7)	NA	509 (10.5)	NA
≥20	6547 (78.1)	NA	3454 (71.5)	NA
Record of autoimmune disease	2309 (27.6)	967 774 (6.8)	1376 (28.5)	927 (10.8)
Age at record of autoimmune disease, y				
0-9	279 (12.1)	70 129 (7.2)	220 (16.0)	84 (9.1)
10-19	235 (10.2)	65 473 (6.8)	185 (13.4)	87 (9.4)
≥20	1795 (77.7)	832 172 (86.0)	971 (70.6)	756 (81.6)

Abbreviations: NA, not applicable; PID, primary humoral immunodeficiency.

^a^ Percentages have been rounded and may not total 100.

### Cohort Analyses

A total of 1720 individuals who had a record of PID (20.5% of all individuals with PID) and 1 524 737 of the unexposed individuals (10.7%) had at least 1 diagnosis of a psychiatric disorder (Table 2). In the fully adjusted model, individuals with PID had a 91% higher likelihood of any psychiatric disorder (adjusted OR [AOR], 1.91 [95% CI, 1.81-2.01]) compared with unexposed individuals. The likelihood of having an individual psychiatric disorder was also significantly higher for most outcomes, with AORs ranging from 1.34 (95% CI, 1.17-1.54) for schizophrenia and other psychotic disorders to 2.99 (95% CI, 2.42-3.70) for autism spectrum disorders. Exposed individuals also had an increased likelihood of any suicidal behavior (AOR, 1.84; 95% CI, 1.66-2.04) and individual outcomes (AOR for death by suicide, 1.84 [95% CI, 1.25-2.71]; AOR for suicide attempts, 1.84 [95% CI, 1.66-2.04]) compared with those who were unexposed (Table 2).

**Table 2.** Associations Between Exposure to PID and Psychiatric Disorders and Suicidal Behavior in the Population Cohort and in the Full-Siblings Subcohort

Disorder^a^	Population cohort				Full-siblings cohort			
	No. (%)		OR (95% CI)		No. (%)		OR (95% CI)	
	PID exposed (n = 8378)	PID unexposed (n = 14 297 937)	Minimally adjusted^b^	Fully adjusted^c^	PID exposed (n = 4828)	PID unexposed (n = 8584)	Minimally adjusted^d^	Fully adjusted^c^
Any psychiatric disorder	1720 (20.5)	1 524 737 (10.7)	2.16 (2.05-2.28)^e^	1.91 (1.81-2.01)^e^	1027 (21.3)	1228 (14.3)	1.71 (1.54-1.89)^e^	1.64 (1.48-1.83)^e^
Autism spectrum disorders	89 (1.1)	53 868 (0.4)	3.50 (2.84-4.32)^e^	2.99 (2.42-3.70)^e^	63 (1.3)	49 (0.6)	2.25 (1.45-3.49)^e^	2.29 (1.43-3.66)^e^
Attention-deficit/hyperactivity disorder	126 (1.5)	114 713 (0.8)	2.31 (1.93-2.76)^e^	1.99 (1.67-2.38)^e^	82 (1.7)	97 (1.1)	1.46 (1.04-2.06)^f^	1.47 (1.03-2.11)^f^
Obsessive-compulsive disorder	54 (0.6)	37 734 (0.3)	2.49 (1.91-3.26)^e^	2.19 (1.68-2.86)^e^	35 (0.7)	40 (0.5)	1.55 (0.94-2.54)	1.55 (0.94-2.56)
Eating disorders	70 (0.8)	40 271 (0.3)	3.03 (2.39-3.84)^e^	2.54 (2.00-3.21)^e^	48 (1.0)	36 (0.4)	1.68 (0.99-2.83)	1.52 (0.86-2.68)
Schizophrenia and other psychotic disorders	204 (2.4)	270 729 (1.9)	1.42 (1.24-1.63)^e^	1.34 (1.17-1.54)^e^	106 (2.2)	171 (2.0)	1.23 (0.94-1.61)	1.23 (0.93-1.63)
Bipolar disorder	100 (1.2)	88 994 (0.6)	1.86 (1.53-2.26)^e^	1.65 (1.35-2.01)^e^	53 (1.1)	80 (0.9)	1.16 (0.80-1.68)	1.08 (0.73-1.59)
Anxiety disorders	777 (9.3)	522 924 (3.7)	2.62 (2.44-2.83)^e^	2.25 (2.09-2.42)^e^	503 (10.4)	519 (6.0)	1.65 (1.43-1.91)^e^	1.61 (1.39-1.87)^e^
Major depression disorder and other mood disorders	870 (10.4)	625 709 (4.4)	2.45 (2.28-2.63)^e^	2.10 (1.95-2.25)^e^	489 (10.1)	538 (6.3)	1.65 (1.43-1.90)^e^	1.50 (1.29-1.74)^e^
Substance use disorders	510 (6.1)	522 981 (3.7)	1.84 (1.68-2.02)^e^	1.62 (1.48-1.77)^e^	326 (6.8)	475 (5.5)	1.50 (1.27-1.77)^e^	1.46 (1.23-1.73)^e^
Any suicidal behavior	399 (4.8)	332 435 (2.3)	2.09 (1.89-2.31)^e^	1.84 (1.66-2.04)^e^	258 (5.3)	338 (3.9)	1.43 (1.19-1.72)^e^	1.37 (1.14-1.66)^g^
Death by suicide	26 (0.3)	25 535 (0.2)	1.90 (1.29-2.79)^g^	1.84 (1.25-2.71)^g^	16 (0.3)	29 (0.3)	0.95 (0.49-1.85)	0.80 (0.39-1.61)
Suicide attempts	382 (4.6)	314 465 (2.2)	2.10 (1.90-2.33)^e^	1.84 (1.66-2.04)^e^	247 (5.1)	317 (3.7)	1.47 (1.22-1.77)^e^	1.41 (1.17-1.71)^e^

Abbreviations: OR, odds ratio; PID, primary humoral immunodeficiency.

^a^ Tourette syndrome or chronic tic disorders are not reported as a separate entity owing to underpowered analysis. Total numbers and percentage of the specific outcomes may not sum to that of the combined outcomes because the study participants may have more than 1 specific outcome.

^b^ Adjusted for individual’s year of birth and sex.

^c^ Adjusted for individual’s year of birth and sex and history of autoimmune disease.

^d^ Adjusted for year of birth and sex for exposed and unexposed siblings.

^e^ *P* < .001, Wald test.

^f^ *P* < .05, Wald test.

^g^ *P* < .01, Wald test.

### Sibling Analyses

A total of 4776 clusters of full siblings discordant for PID were identified, consisting of 4828 individuals with PID and their 8584 unexposed siblings. In the sibling analysis, the strength of the associations was attenuated but remained significant for the aggregate outcomes (AOR for any psychiatric disorder, 1.64 [95% CI, 1.48-1.83]; AOR for any suicidal behavior, 1.37 [95% CI, 1.14-1.66]), many of the individual outcomes (range of AORs, 1.46 [95% CI, 1.23-1.73] for substance use disorders to 2.29 [95% CI, 1.43-3.66] for autism spectrum disorders), and suicide attempts (AOR, 1.41; 95% CI, 1.17-1.71) (Table 2).

### Additional Analyses

#### Single vs Joint Exposure

In the whole study cohort, the associations between exposure to PID only and aggregated outcomes (AOR for any psychiatric disorder, 2.08 [95% CI, 1.95-2.22]; AOR for any suicidal behavior, 1.98 [95% CI, 1.75-2.25]) as well as most individual outcomes (range of AORs, 2.23 [95% CI, 2.04-2.43] for major depression disorder and other mood disorders to 3.47 [95% CI, 2.71-4.45] for autism spectrum disorders) were significantly stronger (nonoverlapping 95% CIs) than the corresponding associations for exposure to autoimmune disease only (Table 3 and Figure). Furthermore, individuals jointly exposed to PID and autoimmune disease had significantly higher ORs for any psychiatric disorders (AOR, 2.77; 95% CI, 2.52-3.05) and any suicidal behaviors (AOR, 2.75; 95% CI, 2.32-3.27) than individuals exposed to either PID only (AORs, 2.08 [95% CI, 1.95-2.22] and 1.98 [95% CI, 1.75-2.25], respectively) or autoimmune disease only (AORs, 1.73 [95% CI, 1.72-1.74] for any psychiatric disorders and 1.71 [95% CI, 1.69-1.73] for any suicidal behavior) (Table 3 and Figure).

**Table 3.** Association Between Exposure to PID Only, AD Only, or PID Plus AD and Psychiatric Disorders and Suicidal Behavior in the Population Cohort

Disorder^a^	Unexposed, No. (%) (n = 13 330 163)	Exposed to PID only		Exposed to AD only		Exposed to PID plus AD	
		No. (%) (n = 6069)	OR (95% CI)^b^	No. (%) (n = 967 774)	OR (95% CI)^b^	No. (%) (n = 2309)	OR (95% CI)^b^
Any psychiatric disorder	1 362 707 (10.2)	1164 (19.2)	2.08 (1.95-2.22)^c^	162 030 (16.7)	1.73 (1.72-1.74)^c^	556 (24.1)	2.77 (2.52-3.05)^c^
Autism spectrum disorders	50 060 (0.4)	64 (1.1)	3.47 (2.71-4.45)^c^	3808 (0.4)	1.90 (1.83-1.96)^c^	25 (1.1)	4.18 (2.81-6.22)^c^
Attention-deficit/hyperactivity disorder	106 702 (0.8)	93 (1.5)	2.35 (1.91-2.89)^c^	8011 (0.8)	1.84 (1.79-1.88)^c^	33 (1.4)	2.53 (1.79-3.58)^c^
Obsessive-compulsive disorder	34 225 (0.3)	43 (0.7)	2.85 (2.11-3.85)^c^	3509 (0.4)	1.68 (1.63-1.74)^c^	11 (0.5)	1.93 (1.07-3.49)^d^
Eating disorders	36 254 (0.3)	44 (0.7)	2.83 (2.10-3.81)^c^	4017 (0.4)	1.96 (1.89-2.02)^c^	26 (1.1)	4.21 (2.86-6.21)^c^
Schizophrenia and other psychotic disorders	245 327 (1.8)	140 (2.3)	1.37 (1.16-1.62)^c^	25 402 (2.6)	1.30 (1.29-1.32)^c^	64 (2.8)	1.67 (1.30-2.15)^c^
Bipolar disorder	78 850 (0.6)	67 (1.1)	1.82 (1.43-2.31)^c^	10 144 (1.0)	1.65 (1.62-1.69)^c^	33 (1.4)	2.29 (1.63-3.23)^c^
Anxiety disorders	465 039 (3.5)	517 (8.5)	2.54 (2.32-2.78)^c^	57 885 (6.0)	1.89 (1.87-1.91)^c^	260 (11.3)	3.40 (2.99-3.87)^c^
Major depression disorder and other mood disorders	547 068 (4.1)	544 (9.0)	2.23 (2.04-2.43)^c^	78 641 (8.1)	1.92 (1.91-1.94)^c^	326 (14.1)	3.61 (3.21-4.06)^c^
Substance use disorders	466 930 (3.5)	360 (5.9)	1.85 (1.67-2.06)^c^	56 051 (5.8)	1.76 (1.75-1.78)^c^	150 (6.5)	2.16 (1.83-2.55)^c^
Any suicidal behavior	296 588 (2.2)	263 (4.3)	1.98 (1.75-2.25)^c^	35 847 (3.7)	1.71 (1.69-1.73)^c^	136 (5.9)	2.75 (2.32-3.27)^c^
Death by suicide	23 624 (0.2)	19 (0.3)	1.90 (1.21-2.99)^e^	1911 (0.2)	1.16 (1.11-1.21)^c^	7 (0.3)	1.96 (0.93-4.12)
Suicide attempts	279 828 (2.1)	252 (4.2)	2.00 (1.77-2.27)^c^	34 637 (3.6)	1.75 (1.73-1.77)^c^	130 (5.6)	2.76 (2.32-3.30)^c^

Abbreviations: AD, autoimmune disease; OR, odds ratio; PID, primary humoral immunodeficiency.

^a^ Tourette syndrome or chronic tic disorders are not reported as a separate entity owing to underpowered analysis. Total numbers and percentage of the specific outcomes may not sum to that of the combined outcomes because the study participants may have more than 1 specific outcome.

^b^ Adjusted for individual’s year of birth and sex.

^c^ *P* < .001, Wald test.

^d^ *P* < .05, Wald test.

^e^ *P* < .01, Wald test.

**Figure.**
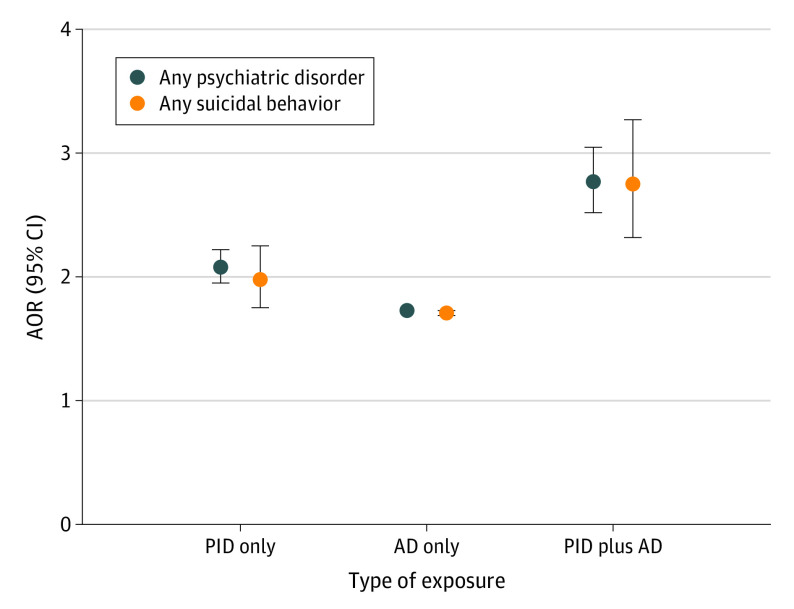
Association of Single and Joint Exposure to Primary Humoral Immunodeficiency (PID) and Autoimmune Disease (AD) With Any Psychiatric Disorder and Any Suicidal Behavior Individuals were categorized into mutually exclusive categories of PID only, AD only, joint exposures (PID plus AD), or no exposure (reference). Error bars for association of AD only with any psychiatric disorder and any suicidal behavior are not visible owing to narrow 95% CIs. AOR indicates adjusted odds ratio.

### Selective IgA Deficiency

In a subcohort of individuals living in Sweden from 1997 onward, 3123 had a record of selective IgA deficiency and were compared with 11 593 012 individuals without any PID. In the analyses of the whole cohort, fully adjusted models revealed increased likelihoods for aggregated outcomes (AOR for any psychiatric disorder, 1.79 [95% CI, 1.64-1.95]; AOR for any suicidal behavior, 1.45 [95% CI, 1.22-1.73]) and all individual outcomes (range of AORs, 1.43 [95% CI, 1.20-1.72] for suicide attempts to 3.27 [95% CI, 2.46-4.35] for autism spectrum disorders) among the exposed, whereas in the sibling analysis, the associations were attenuated but remained significant for any psychiatric disorders (AOR, 1.49; 95% CI, 1.27-1.76), autism spectrum disorders (AOR, 1.94; 95% CI, 1.11-3.41), depression (AOR, 1.35; 95% CI, 1.07-1.70), and anxiety disorders (AOR, 1.43; 95% CI, 1.15-1.77) (eTable 5 in the Supplement).

### Sex-Stratified Analyses

The associations between PID and any psychiatric disorder and any suicidal behavior were significantly larger for women (AORs, 2.18 [95% CI, 2.04-2.33] and 2.44 [95% CI, 1.99-2.52], respectively) than for men (AORs, 1.54 [95% CI, 1.41-1.68] and 1.24 [1.02-1.50], respectively) (eTable 6 in the Supplement). Likewise, women exposed to any PID only or to any PID plus any autoimmune disease had significantly greater likelihood than men of any psychiatric disorder (AORs, 2.42 [95% CI, 2.24-2.63] vs 1.65 [95% CI, 1.48-1.84]) and any suicidal behavior (AORs, 2.43 [95% CI, 2.09-2.82] vs 1.40 [95% CI, 1.12-1.76]), whereas no difference between sexes appeared in association between any autoimmune disease only and aggregated psychiatric outcomes (eTable 7 and eFigure in the Supplement).

### Inpatient and Outpatient Records of Exposure and Outcomes

When excluding all individuals with an exposure and outcome recorded before 2001, the direction and strength of the associations were similar overall (AOR for any psychiatric disorder, 1.88 [95% CI, 1.75-2.02]; AOR for any suicidal behavior, 1.30 [95% CI, 1.07-1.57]; and range of AORs for individual outcomes, 1.15 [95% CI, 0.81-1.61] for schizophrenia and other psychotic disorders to 2.75 [95% CI, 2.13-3.57] for autism spectrum disorders). The associations were less precise owing to the small numbers of cases for some of the outcomes (eTable 8 in the Supplement).

## Discussion

The main finding of the study is that PIDs are significantly associated with a wide range of psychiatric conditions and suicidal behavior, particularly in women. The associations could not be explained by co-occurring autoimmune diseases or by familial confounders shared by siblings. Furthermore, the association with psychiatric disorders and suicidal behavior was markedly stronger for joint exposure to PID and autoimmune disease than for single exposure to any of these disorders, suggesting an additive effect from these immune-related conditions. Overall, this study is the first, to our knowledge, to provide robust evidence of an association between PIDs and a wide range of psychiatric disorders and the first to demonstrate an association between these immune conditions and suicidal behavior.

Multiple aspects of this study are worth noting. First, although PIDs were associated with an increased likelihood of most psychiatric disorders, the strongest association was found with autism spectrum disorders, a finding that remained significant, albeit attenuated, in the sibling comparison. Multiple lines of evidence suggest that immunological disruption may be involved in the etiopathogenesis of autism spectrum disorders, either through altered maternal immune function in utero or through immune disruption after birth.^25^ Although the current findings cannot inform on any pathological interaction between PIDs and autism spectrum disorders, previous reports of increased inflammatory bowel disease^26^ and gut microbiota dysbiosis^27^ in autism spectrum disorders may be related to PIDs because both selective IgA deficiency and common variable immunodeficiency are associated with an increased risk for inflammatory bowel disease and microbiota dysbiosis.^28,29,30^ At present, no controlled studies have reported the rate of PIDs in autism spectrum disorders or any effect of PID on the clinical course of autism spectrum disorders, although further research in this area is warranted based on our data.

Another unique finding is the association of PIDs with suicidal behavior, as well as evidence that individuals with joint exposure to PID and autoimmune disease displayed the highest association with suicidal behavior compared with individuals with single exposures. To our knowledge, this report is the first to delineate an association between immune deficiency and suicidal behavior, although autoimmune diseases, inflammation, and infections have all been previously shown to increase the risk of suicide.^31,32^ The mechanisms underlying these associations deserve further study, and the results suggest that psychiatric screening and behavioral health maintenance may be necessary in patients with PIDs.

In our sex-stratified analyses, women exposed to PID only, but not those exposed to autoimmune disease only, appeared particularly vulnerable to psychopathology, suggesting that sex-specific mechanisms may be at play. These mechanisms require further investigation, and clinicians should be aware that women with PID may be in particular need of careful long-term monitoring of psychiatric disorders and suicidal behavior.

The broader implications of this study are worth considering. Our data indicate that PID is significantly associated with most of the analyzed psychiatric conditions and suicidal behavior, and the associations could not be explained by autoimmune diseases or shared familial confounders. This finding suggests that antibody dysfunction may play a role in psychiatric disorders. However, the mechanisms that may underlie the association between PID and psychiatric outcomes are likely complex and cannot be directly determined by the present study. Two major clinical implications of PID are recurrent infections and an increased risk of autoimmune diseases. It is plausible that the lifetime burden of repeated infections or autoimmune conditions may create significant stress, further increasing the risk of psychopathology. Chronic stress may further increase the risk of developing autoimmune disease,^13^ thus creating a vicious circle. Interestingly, analysis of selective IgA deficiency, a less severe subtype of PID, suggested that the association with psychiatric disorders and suicidal behavior was not exclusive to cases with severe PID; this analysis might indicate that immune dysfunction per se (vs psychological consequences of being chronically or recurrently ill) is associated with psychopathology. The potential for chronic inflammation in patients with PID may also result in an increased risk of psychopathology, as suggested by a study by Isung et al^11^ in which chronic inflammation within the MALT was robustly associated with both psychiatric disorders and suicidal behavior. Finally, the observed associations could be a consequence of the increased susceptibility of patients with PID to central nervous system infections with detrimental neurodevelopmental consequences^33^ and/or secondary to the repeated use of antibiotic treatments with plausible microbial dysbiosis and associated negative cerebral effects.^3,34^ To date, little is known regarding the role of immunoglobulins in neurodevelopment or homeostatic brain function, although the results of the present study suggest that further investigation of the role of the humoral immune system in the development of psychiatric disorders and suicidal behavior is necessary.

### Strengths and Limitations

The main strengths of the present study are the uniquely large, population-based sample of individuals with PID, a rare set of conditions that are routinely diagnosed and verified through laboratory analyses; the use of nationwide Swedish registers with prospective and uniform data collection, which minimizes the risk of selection, recall, and report biases; and the use of a sibling design, which accounts for familial confounding. Furthermore, our specific focus on a PID subtype with lower severity, as well as a comparison with individuals affected by autoimmune diseases alone, strengthens our hypothesis that PIDs per se are associated with the outcomes of interest.

Study limitations are inherent to register-based data. First, the date of the recorded diagnosis of the exposure and the outcomes may not correspond to the actual date of onset. Thus, we could not confidently establish temporality and make use of the longitudinal data in the registers and, instead, chose to report on associations of lifetime diagnoses. We are therefore not making assumptions of directionality or causality. Second, the potential effect from factors such as recurrent infections or other adverse clinical manifestations, as well as potential immune modulation from antibiotics or psychotropic drugs that may contribute to the reported associations, could not be assessed from our data. Third, our ability to test for specificity of the observed associations was limited. It would have been ideal to use a clinically relevant comparison group, such as patients with an early exposure to another chronic disease associated with increased risk of infections and with immune disruption, which is distinct from antibody dysfunction, such as childhood leukemia. Such comparison, however, was not viable because register data on malignant neoplasms were not available to us under the present project. Fourth, because the National Patient Register only includes records from outpatient specialist care since 2001 and no information from primary care, less severe cases may be underestimated. Such bias was in part accounted for through a sensitivity analysis, in which exposures and outcomes were only collected from 2001 and later. In addition, surveillance bias cannot be fully ruled out because individuals with PID are much more likely to have contact with clinicians and thus have higher chances of receiving psychiatric diagnoses. However, many of the measured psychiatric outcomes are serious and require specialist care in their own right.

## Conclusions

Primary humoral immunodeficiencies are associated with a broad range of psychiatric disorders and suicidal behavior, particularly in women, even after controlling for autoimmune diseases, suggesting a role for antibody dysfunction in psychiatric disorders. However, several other mechanisms are possible. The strength of these associations increased when PID and autoimmune conditions were analyzed in tandem, suggesting a multiple-hit scenario. Additional research should explore the underlying mechanisms behind these associations.

## Notes

Supplement. eTable 1. List of *International Classification of Diseases* (*ICD*) Codes to Collect Records of Primary Humoral Immunodeficiencies From the National Patient Register eTable 2. List of *International Classification of Diseases* (*ICD*) Codes to Collect Records of Autoimmune Diseases From the National Patient Register eTable 3. List of Swedish *International Classification of Diseases* (*ICD*) Codes to Collect Records of Psychiatric Disorders From the National Patient Register eTable 4. List of *International Classification of Diseases* (*ICD*) Codes to Define Suicidal Behavior From the National Patient Register and the Cause of Death Register eTable 5. Associations Between Exposure to Selective IgA Deficiency and Psychiatric Disorders and Suicidal Behavior in the Population Cohort and in the Full-Siblings Subcohort eTable 6. Associations of Primary Immunodeficiencies With Psychiatric Disorders and Suicidal Behavior in the Population Cohort Stratified by Sex eTable 7. Associations of Exposure to Primary Immunodeficiencies Only, Autoimmune Diseases Only, and Joint Exposure to Primary Immunodeficiency and Autoimmune Diseases With Psychiatric Disorders and Suicidal Behavior in the Population Cohort Stratified by Sex eTable 8. Associations Between Exposure to Primary Immunodeficiencies and Psychiatric Disorders and Suicidal Behavior in the Population Cohort and Full Sibling Subcohort, After Excluding Individuals Who Received Their Exposure and/or Outcome Diagnoses Before 2001 eFigure. Association of Single and Joint Exposure to Primary Immunodeficiencies and Autoimmune Diseases With Any Psychiatric Disorder and Any Suicidal Behavior, Stratified by Sex eReferences.
