# Supplementary material for: Association of Primary Humoral Immunodeficiencies With Psychiatric Disorders and Suicidal Behavior and the Role of Autoimmune Diseases
Source: JAMA Psychiatry. 2020 Jun 10;77(11):1–9. doi: 10.1001/jamapsychiatry.2020.1260 (PMC7287945; doi:10.1001/jamapsychiatry.2020.1260)
Supplement: Supplement. — eTable 1. List of International Classification of Diseases (ICD) Codes to Collect Records of Primary Humoral Immunodeficiencies From the National Patient Register eTable 2. List of International Classification of Diseases (ICD) Codes to Collect Records of Autoimmune Diseases From the National Patient Register eTable 3. List of Swedish International Classification of Diseases (ICD) Codes to Collect Records of Psychiatric Disorders From the National Patient Register eTable 4. List of International Classification of Diseases (ICD) Codes to Define Suicidal Behavior From the National Patient Register and the Cause of Death Register eTable 5. Associations Between Exposure to Selective IgA Deficiency and Psychiatric Disorders and Suicidal Behavior in the Population Cohort and in the Full-Siblings Subcohort eTable 6. Associations of Primary Immunodeficiencies With Psychiatric Disorders and Suicidal Behavior in the Population Cohort Stratified by Sex eTable 7. Associations of Exposure to Primary Immunodeficiencies Only, Autoimmune Diseases Only, and Joint Exposure to Primary Immunodeficiency and Autoimmune Diseases With Psychiatric Disorders and Suicidal Behavior in the Population Cohort Stratified by Sex eTable 8. Associations Between Exposure to Primary Immunodeficiencies and Psychiatric Disorders and Suicidal Behavior in the Population Cohort and Full Sibling Subcohort, After Excluding Individuals Who Received Their Exposure and/or Outcome Diagnoses Before 2001 eFigure. Association of Single and Joint Exposure to Primary Immunodeficiencies and Autoimmune Diseases With Any Psychiatric Disorder and Any Suicidal Behavior, Stratified by Sex eReferences. [file jamapsychiatry-e201260-s001.pdf]

Supplementary Online Content

Isung J, Williams K, Isomura K, et al. Association of primary humoral immunodeficiencies with psychiatric disorders and suicidal behavior and the role of autoimmune diseases. *JAMA Psychiatry*. Published online June 10, 2020. doi:10.1001/jamapsychiatry.2020.1260

eTable 1. List of *International Classification of Diseases* (*ICD*) Codes to Collect Records of Primary Humoral Immunodeficiencies From the National Patient Register
eTable 2. List of *International Classification of Diseases* (*ICD*) Codes to Collect Records of Autoimmune Diseases From the National Patient Register
eTable 3. List of Swedish *International Classification of Diseases* (*ICD*) Codes to Collect Records of Psychiatric Disorders From the National Patient Register
eTable 4. List of *International Classification of Diseases* (*ICD*) Codes to Define Suicidal Behavior From the National Patient Register and the Cause of Death Register
eTable 5. Associations Between Exposure to Selective IgA Deficiency and Psychiatric Disorders and Suicidal Behavior in the Population Cohort and in the Full-Siblings Subcohort
eTable 6. Associations of Primary Immunodeficiencies With Psychiatric Disorders and Suicidal Behavior in the Population Cohort Stratified by Sex
eTable 7. Associations of Exposure to Primary Immunodeficiencies Only, Autoimmune Diseases Only, and Joint Exposure to Primary Immunodeficiency and Autoimmune Diseases With Psychiatric Disorders and Suicidal Behavior in the Population Cohort Stratified by Sex
eTable 8. Associations Between Exposure to Primary Immunodeficiencies and Psychiatric Disorders and Suicidal Behavior in the Population Cohort and Full Sibling Subcohort, After Excluding Individuals Who Received Their Exposure and/or Outcome Diagnoses Before 2001
eFigure. Association of Single and Joint Exposure to Primary Immunodeficiencies and Autoimmune Diseases With Any Psychiatric Disorder and Any Suicidal Behavior, Stratified by Sex
eReferences.

This supplementary material has been provided by the authors to give readers additional information about their work.

©2020 Isung J et al. *JAMA Psychiatry*.

eTable 1. List of *International Classification of Diseases* (*ICD*) Codes to Collect Records of Primary Humoral Immunodeficiencies From the National Patient Register

**Diagnoses**	**ICD-8 codes**	**ICD-9 codes**	**ICD-10 codes**	**PID n=8 378 (%)[b]**
Hypogammaglobulinaemia	275,10[a]			744 (8.88)
Primary deficiencies of humoral immunity<br>Agammaglobulinemia<br>Hypogammaglobulinemia<br>Common variable immunodeficiency<br>Selective deficiency of immunoglobulin A<br>Selective deficiency of immunoglobulin G		279J[a]		1 469 (17.53)
Hereditary hypogammaglobulinemia			D80.0	2 244 (26.78)
Nonfamilial hypogammaglobulinemia			D80.1	3 519 (42.00)
Selective deficiency of immunoglobulin A [IgA]			D80.2	4 050 (48.34)
Selective deficiency of immunoglobulin G [IgG] subclasses			D80.3	4 786 (57.13)
Selective deficiency of immunoglobulin M [IgM]			D80.4	2 096 (25.02)
Immunodeficiency with increased immunoglobulin M [IgM]			D80.5	57 (0.68)
Common variable immunodeficiency with predominant abnormalities of B-cell numbers and function			D83.0	2 317 (27.66)
Common variable immunodeficiency with predominant immunoregulatory T-cell disorders			D83.1	139 (1.66)
Common variable immunodeficiency with autoantibodies to B- or T-cells			D83.2	2 037 (24.31)
Other common variable immunodeficiency			D83.8	2 130 (25.42)
Common variable immunodeficiency, unspecified			D83.9	2 405 (28.71)

[a] In ICD-8 and 9 there was only one diagnostic code representing primary deficiencies of humoral immunity.
[b] Individual ICD-codes for subtypes of primary immunodeficiency (PID) could only be ascertained in ICD-10. The sum of individuals with a specific PID diagnosis may exceed the total number of PID individuals since patients could have more than one code in the register.

©2020 Isung J et al. *JAMA Psychiatry*.

eTable 2. List of *International Classification of Diseases* (*ICD*) Codes to Collect Records of Autoimmune Diseases From the National Patient Register

Diagnoses	ICD-8 codes	ICD-9 codes	ICD-10 codes
Acute disseminated encephalomyelitis	-	-	G04
Alopecia areata	704.00	704.A	L63
Anti-NMDA receptor encephalitis	-	-	G13.1
Antiphospholipid syndrome	-	-	D68.61
Autoimmune polyglandular syndrome	258.10	258.B	E31.0
Behçet's disease	136.07	136.B	M35.2
Bullous pemphigoid	694.02	694.F	L12.0
Celiac disease	269.10	579.A	K90.0
Churg-Strauss syndrome	-	-	M30.1
Crohn's disease	563.00	555	K50
Dermatomyositis	716.00	710.D	M33.90
Graves' disease	242.00	242.A	E05.0
Guillain-Barré syndrome	354.01	357.A	G61.0
Hashimoto's thyroiditis	245.03	245.C	E06.3
Henoch-Schonlein purpura	287.00	287.A	D69.0
Idiopathic thrombocytopenic purpura	287.10 / 446.38	287.D	D69.3
IgA nephropathy	580 /582	580 /582	N00/N01/N03/N05
Kawasaki	-	446.1	M30.3
Microscopic polyangiitis	-	-	M31.7
Mixed connective tissue disease	734.91	710.W	M35.1
Multiple sclerosis	340	340	G35
Myasthenia gravis	733.00	358.A	G70.0
Pemphigus vulgaris	694	694.E	L10.0
Polymyalgia rheumatica	-	725	M35.3
Polyarteritis nodosa	446.09	446.0	M30.0
Primary biliary cirrhosis	571.90	571.F	K74.3
Psoriasis vulgaris	696.10 / 696.19-23	696	L40
Rheumatic fever	390/391	390/391	I00/I01
Rheumatic fever with chorea	392	392	I02
Rheumatoid arthritis	712.00 / 712.10/ 712.19 / 712.39 / 712.59	714.A	M06
Scarlet fever	034.1	034.1	A38.9
Scleroderma (systemic sclerosis)	734.0 / 734.01 / 734.09	710.B	M34
Sjögren's syndrome	734.90	710.C	M35.0
Systemic Lupus Erythematosus	734.10	710.A	M32
Temporal arteritis	446.30	446.F	M31.5
Thrombotic thrombocytopenic purpura	446.40	446.G	M31.1
Type 1 diabetes mellitus	-	-	E10.9
Ulcerative colitis	563.10	556	K51
Vitiligo	709.05	709.A	L80
Wegener's granulomatosis	446.20	446.E	M31.3

©2020 Isung J et al. *JAMA Psychiatry*.

eTable 3. List of Swedish *International Classification of Diseases* (*ICD*) Codes to Collect Records of Psychiatric Disorders From the National Patient Register

Diagnoses	ICD-8 codes	ICD-9 codes	ICD-10 codes	Age constrains	Median age (IQR) of first record of diagnosis in the registers, years
Autism spectrum disorders	-	299A	F84.0, F84.1, F84.8, F84.9	If recorded at age ≥1 year	16.39 (10.21-26.06)
Attention-deficit/hyperactivity disorder<br>Diagnosis defined through both ICD-code or prescription of ADHD drugs[a] as recorded in the Prescribed Drug Register. The validity of this definition has been previously described.[1]	-	314W, 314X	F90<br>Prescribed Drug Register codes:[a] N06BA01, N06BA02, N06BA04,<br>N06BA09,<br>N06BA12	If recorded at age ≥3 years	17.29 (11.60-29.12)
Tourette's and chronic tic disorders	306.2 following the algorithm described elsewhere.[2]	307C following the algorithm described elsewhere.[2]	F95.1, F95.2 following the algorithm described elsewhere.[2]	If recorded at age ≥3 years	13.90 (10.36-22.41)
Obsessive-compulsive disorder	300.3	300D	F42	If recorded at age 6 years or above	28.92 (20.13-41.67)

©2020 Isung J et al. *JAMA Psychiatry*.

Eating disorders	-	307B, 307F	F50.0/.1/.2/.3/.8/.9	If recorded at age ≥8 years	19.80 (16.24-26.44)
Schizophrenia and other psychotic disorders	291, 295, 296.99, 297, 298, 299	291, 292, 295, 296X, 297, 298, 299 (except for 299A)	F10-F19 subsection .5, F20, F21, F22, F23, F24, F25, F28, F29, F32.3	If recorded at age ≥10 years	50.60 (33.75-70.44)
Bipolar disorder	296, except 296.2 and 296.99	296, except 296B and 296X	F30, F31	If recorded at age ≥10 years	44.70 (31.04-59.78)
Anxiety disorders	3000, 3002	300A, 300C, 308,309	F40.0, F40.1, F41.1	If recorded at age ≥6 years	38.63 (25.57-53.27)
Major depression disorder and other mood disorders	296.2, 300.4	296B, 300E, 311	F32 (except for F32.3), F33, F34, F38, F39	If recorded at age ≥10 years	45.87 (30.13-64.66)
Substance use disorders	303, 304	303, 304, 305A, 305X	F10-F19 (except subsection .5)	If recorded at age ≥10 years	41.92 (26.32-56.63)

[a] In the Prescribed Drug Register, drug classes are defined following the Anatomical Therapeutic Chemical (ATC) classification system codes.
Abbreviation: IQR, Interquartile range

eTable 4. List of *International Classification of Diseases* (*ICD*) Codes to Define Suicidal Behavior From the National Patient Register and the Cause of Death Register

Diagnoses[c]	ICD codes[a]	Median age (IQR) of first record of diagnosis in the registers, years	
		**Deaths by suicide**	**Suicide attempts**
Suicide and self-inflicted injury	E950-E959 (ICD-8 and ICD-9), if recorded at age ≥10 years	51.29 (37.22-64.46)	34.42 (22.12-51.13)
Intentional self-harm	X60-X84 (ICD-10), if recorded at age ≥10 years		
Injury undetermined whether accidental or purposely inflicted	E980-E989 (ICD-8 and ICD-9), if recorded at age ≥10 years		
Events of undetermined intent	Y10-Y34 (ICD-10), if recorded at age ≥10 years		

[a] Diagnoses of suicide attempts were retrieved from the National Patient Register and were coded according to the Swedish version of ICD-codes in its 8th, 9th, or 10th revision.

[b] Diagnoses of death by suicide were retrieved from the Cause of Death Register using the same codes as for suicide attempts and were coded according to the International version of ICD-codes in its 8th, 9th, or 10th revision.

[c] Consistent with previous suicide research, both certain and undetermined causes were included in the definition of suicidal behavior.[3,4]

Abbreviation: IQR, Interquartile range

©2020 Isung J et al. *JAMA Psychiatry*.

eTable 5. Associations Between Exposure to Selective IgA Deficiency and Psychiatric Disorders and Suicidal Behavior in the Population Cohort and in the Full-Siblings Subcohort

	**Population cohort**				**Full siblings cohort**			
	**No. (%)**		**OR (95% CI)**		**No. (%)**		**OR (95% CI)**	
	**Exposed (n=3 123)**	**Unexposed (n=11 593 012)**	**Minimally adjusted[a]**	**Fully adjusted[b]**	**Exposed (n=2 019)**	**Unexposed (n=3 417)**	**Minimally adjusted[c]**	**Fully adjusted[b]**
**Any psychiatric disorder[d]**	644 (20.62)	1 308 053 (11.28)	**2.03 (1.86-2.21)*****	**1.79 (1.64-1.95)*****	411 (20.39)	501 (14.66)	**1.62 (1.38-1.90)*****	**1.49 (1.27-1.76)*****
Autism spectrum disorders	49 (1.57)	53 806 (0.46)	**3.88 (2.92-5.16)*****	**3.27 (2.46-4.35)*****	39 (1.93)	34 (1.00)	**2.13 (1.21-3.73)****	**1.94 (1.11-3.41)***
Attention-deficit/hyperactivity disorder	63 (2.02)	135 427 (1.17)	**1.95 (1.52-2.51)*****	**1.65 (1.28-2.13)*****	41 (2.03)	57 (1.67)	1.22 (0.76-1.98)	1.13 (0.70-1.84)
Obsessive-compulsive disorder	25 (0.80)	36 370 (0.31)	**2.57 (1.73-3.81)*****	**2.27 (1.53-3.37)*****	16 (0.79)	18 (0.53)	1.55 (0.77-3.15)	1.50 (0.74-3.04)
Eating disorders	23 (0.74)	39 253 (0.34)	**2.18 (1.45-3.29)*****	**1.81 (1.20-2.73)****	18 (0.89)	18 (0.53)	0.99 (0.43-2.28)	0.89 (0.38-2.08)
Schizophrenia and other psychotic disorders	69 (2.21)	163 963 (1.41)	**1.62 (1.28-2.06)*****	**1.53 (1.20-1.94)*****	38 (1.88)	52 (1.52)	1.33 (0.84-2.08)	1.26 (0.80-1.98)
Bipolar disorder	39 (1.25)	73 873 (0.64)	**1.90 (1.38-2.60)*****	**1.67 (1.22-2.30)****	20 (0.99)	29 (0.85)	1.30 (0.71-2.41)	1.23 (0.66-2.27)
Anxiety disorders	299 (9.57)	511 318 (4.41)	**2.23 (1.98-2.51)*****	**1.95 (1.73-2.20)*****	210 (10.42)	234 (6.85)	**1.54 (1.25-1.91)*****	**1.43 (1.15-1.77)****
Major depression disorder and other mood disorders	310 (9.93)	551 594 (4.76)	**2.14 (1.90-2.40)*****	**1.85 (1.64-2.08)*****	184 (9.13)	220 (6.44)	**1.49 (1.19-1.88)*****	**1.35 (1.07-1.70)***
Substance use disorders	176 (5.64)	433 036 (3.74)	**1.62 (1.39-1.89)*****	**1.42 (1.22-1.66)*****	125 (6.20)	179 (5.24)	**1.38 (1.06-1.81)***	1.30 (0.99-1.70)
**Any suicidal behavior[d]**	128 (4.10)	293 298 (2.53)	**1.63 (1.37-1.95)*****	**1.45 (1.22-1.73)*****	89 (4.41)	138 (4.04)	1.16 (0.86-1.55)	1.11 (0.82-1.49)
Death by suicide	12 (0.38)	24 967 (0.22)	**1.96 (1.11-3.46)***	**2.02 (1.14-3.56)***	7 (0.35)	10 (0.29)	1.26 (0.44-3.63)	1.29 (0.45-3.73)
Suicide attempt	121 (3.87)	275 715 (2.38)	**1.63 (1.36-1.96)*****	**1.43 (1.20-1.72)*****	84 (4.17)	132 (3.86)	1.13 (0.83-1.53)	1.08 (0.79-1.46)

Note: Significant ORs are marked in bold. Tourette syndrome or chronic tic disorders are not reported as a separate entity due to underpowered analysis.

[a] Adjusted for individual's year of birth and sex.

[b] Additionally adjusted for history of autoimmune disease.

[c] Adjusted for year of birth and sex on both exposed and unexposed siblings.

[d] Total numbers and percentage of the specific outcomes may not sum up to that of the combined outcomes as the study participants may have more than one specific outcome.

* p<0.05, ** p<0.01, *** p<0.001

Abbreviation: OR, odds ratio; CI, confidence intervals.

©2020 Isung J et al. *JAMA Psychiatry*.

eTable 6. Associations of Primary Immunodeficiencies With Psychiatric Disorders and Suicidal Behavior in the Population Cohort Stratified by Sex

	Population cohort			
	No. (%)		OR (95% CI)	
**Men**<br>**Women**	**Exposed (n=3 431) (n=4 947)**	**Unexposed (n=7 219 283) (n=7 078 654)**	**Minimally adjusted[a]**	**Fully adjusted OR[b]**
**Any psychiatric disorder**				
Men	569 (16.58)	753 763 (10.44)	**1.73 (1.58-1.89)*****	**1.54 (1.41-1.68)*****
Women	1 151 (23.27)	770 974 (10.89)	**2.48 (2.32-2.65)*****	**2.18 (2.04-2.33)*****
**Autism spectrum disorders**				
Men	61 (1.78)	36 517 (0.51)	**3.71 (2.87-4.79)*****	**3.20 (2.47-4.13)*****
Women	28 (0.57)	17 351 (0.25)	**3.05 (2.10-4.43)*****	**2.58 (1.78-3.75)*****
**Attention-deficit/hyperactivity disorder**				
Men	68 (1.98)	73 260 (1.01)	**2.04 (1.60-2.61)*****	**1.79 (1.41-2.29)*****
Women	58 (1.17)	41 453 (0.59)	**2.62 (2.02-3.40)*****	**2.22 (1.71-2.88)*****
**Obsessive-compulsive disorder**				
Men	21 (0.61)	16 399 (0.23)	**2.65 (1.73-4.08)*****	**2.37 (1.54-3.64)*****
Women	33 (0.67)	21 335 (0.30)	**2.41 (1.71-3.39)*****	**2.10 (1.49-2.96)*****
**Eating disorders**				
Men	5 (0.15)	3 163 (0.04)	**3.29 (1.37-7.90)****	**2.76 (1.15-6.65)***
Women	65 (1.31)	37 108 (0.52)	**3.03 (2.37-3.88)*****	**2.53 (1.98-3.24)*****
**Schizophrenia and other psychotic disorders**				
Men	74 (2.16)	141 710 (1.96)	1.21 (0.96-1.53)	1.15 (0.91-1.45)
Women	130 (2.63)	129 019 (1.82)	**1.58 (1.33-1.88)*****	**1.49 (1.25-1.78)*****
**Bipolar disorder**				

©2020 Isung J et al. *JAMA Psychiatry*.

Men	24 (0.70)	34 740 (0.48)	**1.50 (1.01-2.24)***	1.33 (0.89-1.98)
Women	76 (1.54)	54 254 (0.77)	**2.02 (1.61-2.53)*****	**1.79 (1.43-2.24)*****
**Anxiety disorders**				
Men	201 (5.86)	202 483 (2.80)	**2.12 (1.84-2.45)*****	**1.83 (1.59-2.11)*****
Women	576 (11.64)	320 441 (4.53)	**2.89 (2.65-3.15)*****	**2.46 (2.25-2.68)*****
**Major depression disorder and other mood disorders**				
Men	229 (6.67)	244 679 (3.39)	**2.08 (1.82-2.38)*****	**1.79 (1.56-2.04)*****
Women	641 (12.96)	381 030 (5.38)	**2.62 (2.41-2.85)*****	**2.24 (2.06-2.44)*****
**Substance use disorders**				
Men	202 (5.89)	350 599 (4.86)	**1.26 (1.09-1.45)****	1.12 (0.97-1.29)
Women	308 (6.23)	172 382 (2.44)	**2.68 (2.38-3.00)*****	**2.28 (2.03-2.56)*****
**Any suicidal behavior**				
Men	106 (3.09)	163 058 (2.26)	**1.38 (1.14-1.68)****	**1.24 (1.02-1.50)***
Women	293 (5.92)	169 377 (2.39)	**2.58 (2.29-2.90)*****	**2.24 (1.99-2.52)*****
**Death by suicide**				
Men	8 (0.23)	17 996 (0.25)	0.95 (0.47-1.90)	0.93 (0.46-1.86)
Women	18 (0.36)	7 539 (0.11)	**3.44 (2.16-5.45)*****	**3.28 (2.07-5.22)*****
**Suicide attempt**				
Men	99 (2.89)	149 491 (2.07)	**1.41 (1.15-1.72)*****	**1.25 (1.02-1.52)***
Women	283 (5.72)	164 974 (2.33)	**2.57 (2.28-2.89)*****	**2.21 (1.96-2.49)*****

Note: Significant ORs are marked in bold. Tourette syndrome or chronic tic disorders are not reported as a separate entity due to underpowered analysis.
[a] Adjusted for individual's year of birth and sex.
[b] Additionally adjusted for history of autoimmune disease.
* p<0.05, ** p<0.01, *** p<0.001

©2020 Isung J et al. *JAMA Psychiatry*.

eTable 7. Associations of Exposure to Primary Immunodeficiencies Only, Autoimmune Diseases Only, and Joint Exposure to Primary Immunodeficiency and Autoimmune Diseases With Psychiatric Disorders and Suicidal Behavior in the Population Cohort Stratified by Sex

	**PID only Men n=2 606 Women n=3 463**		**AD only Men n=413 686 Women n=553 878**		**PID and AD Men n=825 Women n=1 484**		**Neither PID or AD Men n=6 805 387 Women n=6 524 776**
	**No. (%)**	**OR (95% CI)**	**No. (%)**	**OR (95% CI)**	**No. (%)**	**OR (95% CI)**	**No. (%)**
**Any psychiatric disorder**							
Men	404 (15.50)	**1.65 (1.48-1.84)*** **	68 257 (16.49)	**1.73 (1.71-1.74)*** **	165 (20.00)	**2.25 (1.90-2.67)*** **	685 506 (10.07)
Women	760 (21.95)	**2.42 (2.24-2.63)*** **	93 773 (16.93)	**1.74 (1.73-1.75)**	391 (26.35)	**3.08 (2.75-3.46)**	677 201 (10.38)
**Autism spectrum disorders**							
Men	45 (1.73)	**3.65 (2.71-4.91)*** **	2 327 (0.56)	**1.89 (1.81-1.98)*** **	16 (1.94)	**4.46 (2.71-7.35)*** **	34 190 (0.50)
Women	19 (0.55)	**3.04 (1.93-4.78)*** **	1 481 (0.27)	**1.88 (1.78-1.98)*** **	9 (0.61)	**3.66 (1.89-7.05)*** **	15 870 (0.24)
**Attention-deficit/hyperactivity disorder**							
Men	55 (2.11)	**2.21 (1.68-2.89)*** **	4 407 (1.06)	**1.78 (1.73-1.84)*** **	13 (1.58)	**1.76 (1.01-3.06)* **	68 853 (1.01)
Women	38 (1.10)	**2.53 (1.83-3.48)*** **	3 604 (0.65)	**1.88 (1.81-1.95)*** **	20 (1.35)	**3.37 (2.16-5.25)*** **	37 849 (0.58)
**Obsessive-compulsive disorder**							
Men	16 (0.61)	**2.74 (1.67-4.47)*** **	1 279 (0.31)	**1.66 (1.57-1.76)*** **	5 (0.61)	**2.75 (1.14-6.62)* **	15 120 (0.22)
Women	27 (0.78)	**2.93 (2.01-4.28)*** **	2 230 (0.40)	**1.70 (1.63-1.78)*** **	6 (0.40)	1.55 (0.69-3.45)	19 105 (0.29)
**Eating disorders**							
Men	4 (0.15)	**3.59 (1.35-9.59)* **	283 (0.07)	**2.04 (1.80-2.31)*** **	1 (0.12)	2.91 (0.41-20.7)	2 880 (0.04)
Women	40 (1.16)	**2.78 (2.04-3.81)*** **	3 734 (0.67)	**1.96 (1.89-2.03)*** **	25 (1.68)	**4.33 (2.91-6.43)*** **	33 374 (0.51)
**Schizophrenia and other psychotic disorders**							
Men	58 (2.23)	1.28 (0.98-1.66)	11 440 (2.76)	**1.33 (1.30-1.36)*** **	16 (1.94)	1.12 (0.68-1.84)	130 270 (1.91)
Women	82 (2.37)	**1.45 (1.17-1.81)*** **	13 962 (2.52)	**1.28 (1.26-1.30)*** **	48 (3.23)	**2.02 (1.51-2.69)*** **	115 057 (1.76)

©2020 Isung J et al. *JAMA Psychiatry*.

**Bipolar disorder**							
Men	17 (0.65)	1.45 (0.90-2.34)	3 428 (0.83)	**1.73 (1.67-1.79)***	7 (0.85)	1.89 (0.90-3.99)	31 312 (0.46)
Women	50 (1.44)	**1.99 (1.50-2.63)***	6 716 (1.21)	**1.62 (1.58-1.66)***	26 (1.75)	**2.43 (1.65-3.57)***	47 538 (0.73)
**Anxiety disorders**							
Men	138 (5.30)	**1.99 (1.68-2.37)***	19 266 (4.65)	**1.94 (1.91-1.97)***	63 (7.64)	**3.00 (2.32-3.88)***	183 217 (2.69)
Women	379 (10.94)	**2.84 (2.55-3.16)***	38 619 (6.97)	**1.87 (1.85-1.89)***	197 (13.27)	**3.58 (3.08-4.16)***	281 822 (4.32)
**Major depression disorder and other mood disorders**							
Men	146 (5.60)	**1.82 (1.54-2.16)***	26 662 (6.44)	**2.01 (1.98-2.03)***	83 (10.06)	**3.44 (2.74-4.32)***	218 017 (3.20)
Women	398 (11.49)	**2.44 (2.19-2.70)***	51 979 (9.38)	**1.88 (1.86-1.90)***	243 (16.37)	**3.67 (3.20-4.21)***	329 051 (5.04)
**Substance use disorders**							
Men	157 (6.02)	**1.34 (1.14-1.58)***	33 654 (8.13)	**1.73 (1.71-1.75)***	45 (5.45)	1.21 (0.89-1.63)	316 945 (4.66)
Women	203 (5.86)	**2.67 (2.32-3.08)***	22 397 (4.04)	**1.86 (1.83-1.89)***	105 (7.08)	**3.28 (2.69-4.00)***	149 985 (2.30)
**<u>Any suicidal behavior</u>**							
Men	79 (3.03)	**1.40 (1.12-1.76)****	14 801 (3.58)	**1.66 (1.64-1.69)***	27 (3.27)	**1.52 (1.04-2.23)***	148 257 (2.18)
Women	184 (5.31)	**2.43 (2.09-2.82)***	21 046 (3.80)	**1.75 (1.72-1.77)***	109 (7.35)	**3.44 (2.83-4.18)***	148 331 (2.27)
**Death by suicide**							
Men	5 (0.19)	0.79 (0.33-1.89)	1187 (0.29)	**1.13 (1.06-1.20)***	3 (0.36)	1.50 (0.48-4.65)	16 809 (0.25)
Women	14 (0.40)	**3.87 (2.29-6.54)***	724 (0.13)	**1.22 (1.13-1.32)***	4 (0.27)	2.57 (0.96-6.86)	6 815 (0.10)
**Suicide attempt**							
Men	74 (2.84)	**1.44 (1.14-1.81)****	13974 (3.38)	**1.73 (1.70-1.76)***	25 (3.03)	**1.54 (1.03-2.29)***	135 517 (1.99)
Women	178 (5.14)	**2.41 (2.08-2.81)***	20663 (3.73)	**1.77 (1.74-1.79)***	105 (7.08)	**3.40 (2.79-4.15)***	144 311 (2.21)

Note: Significant ORs are marked in bold. Tourette syndrome or chronic tic disorders are not reported as a separate entity due to underpowered analysis.
AD autoimmune disease; PID primary immunodeficiency.
* p<0.05, ** p<0.01, *** p<0.001

©2020 Isung J et al. *JAMA Psychiatry*.

eTable 8. Associations Between Exposure to Primary Immunodeficiencies and Psychiatric Disorders and Suicidal Behavior in the Population Cohort and Full Sibling Subcohort, After Excluding Individuals Who Received Their Exposure and/or Outcome Diagnoses Before 2001

	**Population cohort**				**Full siblings cohort**			
	**No. (%)**		**OR (95% CI)**		**No. (%)**		**OR (95% CI)**	
	**Exposed (n=5 440)**	**Unexposed (n= 10 642 431)**	**Minimally adjusted[a]**	**Fully adjusted[b]**	**Exposed (n=3 452)**	**Unexposed (n= 7 708)**	**Minimally adjusted[c]**	**Fully adjusted[b]**
**Any psychiatric disorder[d]**	878 (16.14)	888 878 (8.35)	**2.08 (1.94-2.24)*** **	**1.88 (1.75-2.02)*** **	545 (15.79)	789 (10.24)	**1.70 (1.47-1.96)*** **	**1.64 (1.42-1.90)*** **
Autism spectrum disorders	59 (1.08)	47 299 (0.44)	**3.18 (2.46-4.12)*** **	**2.75 (2.13-3.57)*** **	44 (1.27)	44 (0.57)	**2.14 (1.24-3.69)** **	**2.13 (1.20-3.78)* **
Attention-deficit/hyperactivity disorder	86 (1.58)	102 972 (0.97)	**2.10 (1.70-2.61)*** **	**1.86 (1.50-2.30)*** **	54 (1.56)	81 (1.05)	1.48 (0.96-2.28)	1.50 (0.95-2.35)
Obsessive-compulsive disorder	30 (0.55)	27 478 (0.26)	**2.31 (1.62-3.31)*** **	**2.09 (1.46-2.99)*** **	20 (0.58)	26 (0.34)	1.48 (0.72-3.06)	1.63 (0.77-3.47)
Eating disorders	21 (0.39)	29 384 (0.28)	1.53 (1.00-2.36)	1.30 (0.85-2.01)	15 (0.43)	25 (0.32)	0.72 (0.32-1.64)	0.57 (0.22-1.48)
Schizophrenia and other psychotic disorders	33 (0.61)	52 019 (0.49)	1.21 (0.86-1.71)	1.15 (0.81-1.61)	19 (0.55)	51 (0.66)	0.76 (0.42-1.38)	0.82 (0.44-1.54)
Bipolar disorder	31 (0.57)	37 031 (0.35)	**1.60 (1.12-2.28)** **	**1.46 (1.03-2.08)* **	16 (0.46)	41 (0.53)	1.04 (0.52-2.07)	1.03 (0.51-2.10)
Anxiety disorders	447 8.22)	410 372 (3.86)	**2.16 (1.96-2.38)*** **	**1.97 (1.79-2.17)*** **	288 (8.34)	386 (5.01)	**1.59 (1.31-1.94)*** **	**1.60 (1.30-1.96)*** **
Major depression disorder and other mood disorders	416 (7.65)	364 140 (3.42)	**2.18 (1.97-2.41)*** **	**1.95 (1.77-2.16)*** **	243 (7.04)	335 (4.35)	**1.74 (1.42-2.15)*** **	**1.58 (1.27-1.97)*** **
Substance use disorders	197 (3.62)	244 020 (2.29)	**1.66 (1.44-1.91)*** **	**1.50 (1.30-1.73)*** **	125 (3.62)	249 (3.23)	**1.40 (1.08-1.83)* **	**1.34 (1.02-1.75)* **
**Any suicidal behavior[d]**	108 (1.99)	152 847 (1.44)	**1.41 (1.16-1.70)*** **	**1.30 (1.07-1.57)** **	72 (2.09)	151 (1.96)	1.05 (0.76-1.46)	1.02 (0.73-1.44)
Suicide attempt	104 (1.91)	142 579 (1.34)	**1.45 (1.20-1.76)*** **	**1.33 (1.09-1.61)** **	69 (2.00)	140 (1.82)	1.10 (0.78-1.54)	1.09 (0.77-1.55)

Note: The analyses are based on the individuals who were alive and living in Sweden in 2001-onwards, i.e., when data from both outpatient and inpatient records were available in the National Patient Register. Diagnoses of PID (exposure) and psychiatric disorders and suicidal behavior (outcomes) were considered for analysis if recorded in the National Patient Register in 2001 or later. Significant ORs are marked in bold. Tourette syndrome or chronic tic disorders and death by suicide are not reported as separate entities due to underpowered analyses.

[a] Adjusted for individual's year of birth and sex.

[b] Additionally adjusted for history of autoimmune disease.

[c] Adjusted for year of birth and sex on both exposed and unexposed siblings.

[d] Total numbers and percentage of the specific outcomes may not sum up to that of the combined outcomes as the study participants may have more than one specific outcome.

* p<0.05, ** p<0.01, *** p<0.001

Abbreviation: OR, odds ratio; CI, confidence intervals.

©2020 Isung J et al. *JAMA Psychiatry*.

eFigure. Association of Single and Joint Exposure to Primary Immunodeficiencies and Autoimmune Diseases With Any Psychiatric Disorder and Any Suicidal Behavior, Stratified by Sex

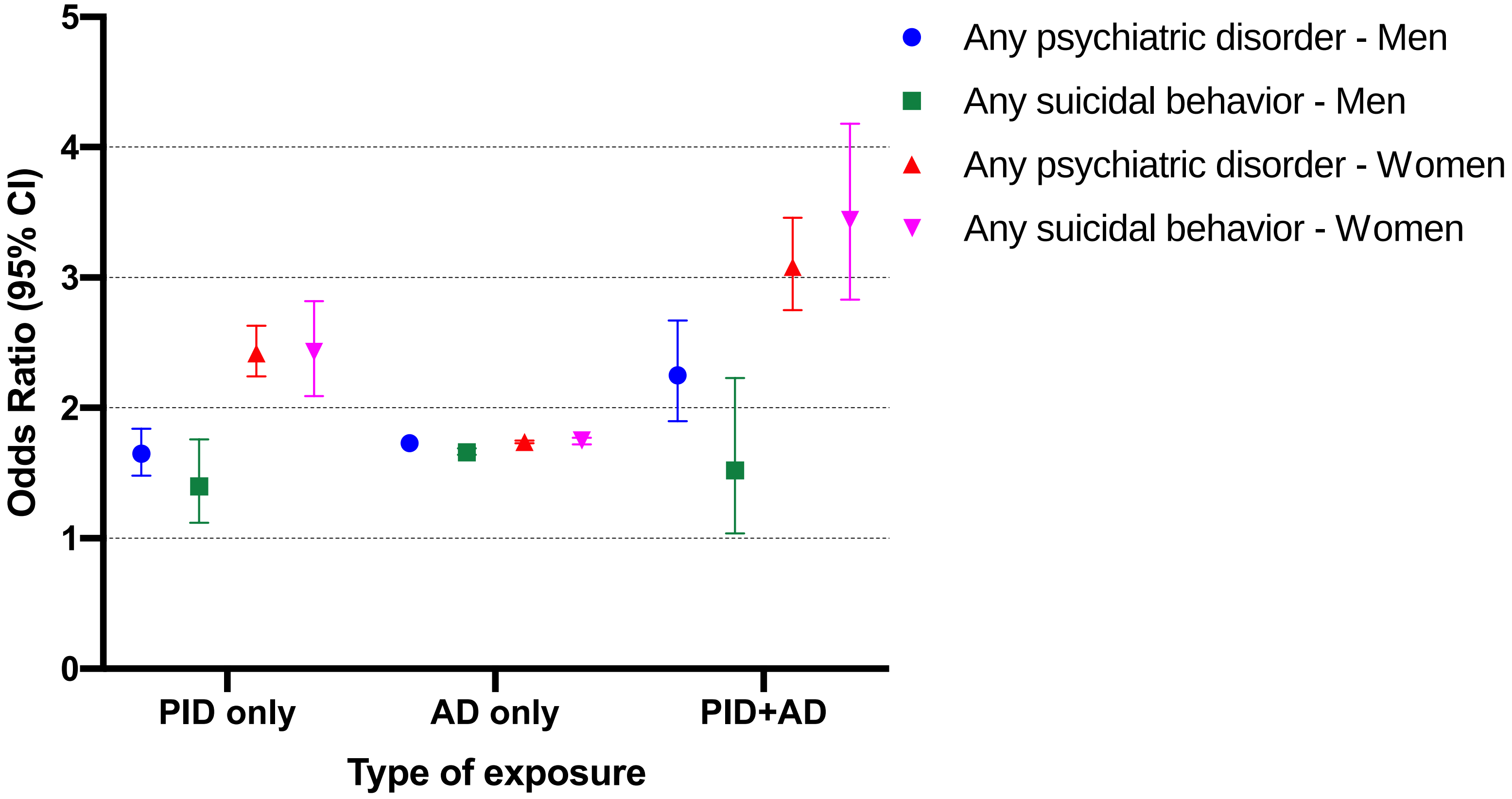

Note: Error bars for association of autoimmune diseases only (AD only) with any psychiatric disorder and any suicidal behavior for men and women are not visible due to narrow confidence intervals.

©2020 Isung J et al. *JAMA Psychiatry*.

eReferences.

1 Larsson H, Rydén E, Boman M, Långström N, Lichtenstein P, Landén M. Risk of bipolar disorder and schizophrenia in relatives of people with attention-deficit hyperactivity disorder. *Br J Psychiatry J Ment Sci* 2013; **203**: 103–106.

2 Rück C, Larsson KJ, Lind K, Perez-Vigil A, Isomura K, Sariaslan A *et al.* Validity and reliability of chronic tic disorder and obsessive-compulsive disorder diagnoses in the Swedish National Patient Register. *BMJ Open* 2015; **5**: e007520.

3 Runeson B, Tidemalm D, Dahlin M, Lichtenstein P, Långström N. Method of attempted suicide as predictor of subsequent successful suicide: national long term cohort study. *BMJ* 2010; **341**: c3222.

4 Fernández de la Cruz L, Rydell M, Runeson B, D'Onofrio BM, Brander G, Rück C *et al.* Suicide in obsessive-compulsive disorder: a population-based study of 36 788 Swedish patients. *Mol Psychiatry* 2016. doi:10.1038/mp.2016.115.

©2020 Isung J et al. *JAMA Psychiatry*.
